# Dynamics of Phytoplankton Communities and Their Characteristics of Realized Niches in a Drinking Reservoir

**DOI:** 10.1002/ece3.71180

**Published:** 2025-04-11

**Authors:** Shuhua Wang, Xujun Liang, Shanshan Zhang, Mingjiang Cai, Zhangxian Xie, Lizhen Lin, Zhenguo Chen, Yiyong Rao, Yanping Zhong

**Affiliations:** ^1^ Key Laboratory of Rural Environmental Remediation and Waste Recycling, College of Resources and Environmental Sciences Quanzhou Normal University Quanzhou Fujian China; ^2^ Guangdong Provincial Engineering Research Center of Intelligent Low‐Carbon Pollution Prevention and Digital Technology, South China Normal University Guangzhou China; ^3^ SCNU (NAN'AN) Green and Low‐Carbon Innovation Center Nan'an SCNU Institute of Green and Low‐Carbon Research Quanzhou China; ^4^ College of Natural Resources and Environment Northwest A&F University Yangling Shanxi China; ^5^ State Key Laboratory of Marine Environmental Science; Fujian Provincial Key Laboratory of Coastal Ecology and Environmental Studies; Taiwan Strait Marine Ecosystem Research Station, Ministry of Education; College of the Environment and Ecology Xiamen University Xiamen China; ^6^ South China Sea Fisheries Research Institute Chinese Academy of Fishery Sciences/Guangdong Provincial Key Laboratory of Fishery Ecology and Environment Guangzhou China

**Keywords:** Cyanophyta, drinking water reservoir, mean niche, niche breadth, phytoplankton community

## Abstract

Realized niches are crucial in defining their optimal conditions and serve as valuable tools for predicting the phytoplankton dynamics in relation to eutrophication, climate change, and harmful phytoplankton blooms. However, previous studies have largely focused on marine ecosystems, leaving freshwater systems less studied. In this study, we elucidate the patterns of phytoplankton community succession based on their niche characteristics in the Shanmei (SM) Reservoir, a drinking water source in Quanzhou, Fujian Province. Additionally, variations in phytoplankton were mainly explained by their realized niche. In the SM Reservoir, total chlorophyll *a* concentrations ranged from 252 to 24,008 ng/L. The phytoplankton community was dominated by Chlorophyta and Cyanophyta, which consisted mostly of *Pseudanabaena* and *Microcystis*, especially in summer. This dominance was attributed to their wide niche breadth and high mean niche for temperature, nitrogen, and dissolved reactive phosphorus. On the other hand, Cryptophyta and Bacillariophyta reached higher concentrations in autumn and winter, linked to their low mean temperature niches. Under the multiple pressures of climate change and anthropogenic activities, Chlorophyta and Cyanophyta are likely to thrive in environments characterized by rising water temperatures and elevated nutrient concentrations. This is particularly true for buoyant cyanobacteria such as *Pseudanabaena,* which are well‐suited to the stratified water layers induced by higher water temperatures. Therefore, incorporating niche characteristics of harmful bloom‐forming species would contribute to the prevention and management of harmful phytoplankton blooms, ultimately improving the safety of drinking water.

## Graphical abstract

The realized niches in water temperature, pH, DO, and nutrient concentrations contribute to explaining the distribution patterns of the phytoplankton community in a drinking water reservoir. Realized niches contribute to understanding phytoplankton dynamics, offering valuable insights for the scientific management and prevention of phytoplankton blooms in reservoirs.

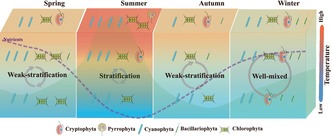

**Abbreviations**
Baci: Bacillariophyta
CCA: canonical correspondence analysis
Chlo: Chlorophyta
Cryp: Cryptophyta
Cyan: Cyanophyta
DO: dissolved oxygen
DRP: dissolved reactive phosphorus
DSi: dissolved silicate
GAMs: generalized additive models
HPLC‐CHEMTAX: high‐performance liquid chromatography coupled with CHEMTAX
MaxEnt: maximum entropy
NO_3_‐N: nitrate NO_2_‐N; nitrite
NO_X_
: the sum of nitrate and nitrite
PCA: principal component analysis.
Pyrr: Pyrrophyta
SM Reservoir: Shanmei Reservoir
TChl *a*
: total Chlorophyll *a*

## 1 Introduction

Drinking water reservoirs, formed by the construction of dams, embankments, and other engineering structures in river channels, play a vital role in ensuring a reliable supply of safe drinking water, particularly in regions facing water scarcity or growing demand (Zhang et al. 2024). In China, significant economic and social pressures have resulted in the development of approximately 100,000 reservoirs (Song et al. 2022), and lakes and reservoirs serve as the water source for 40.8% of the 3441 centralized drinking water sources located in all provinces (Zhang et al. 2024). However, intensive human activities, such as land use changes and alterations to watershed hydrology, have contributed to substantial nutrient influx into these reservoirs. This has resulted in the retention, sedimentation, and enrichment of various nutrients, heightening the occurrences of algal blooms and deteriorating water quality (Ruan et al. 2024). Furthermore, the exacerbation of eutrophication, combined with the persistent effects of climate change, is accelerating shifts in freshwater ecosystems (Cai et al. 2024). These changes underscore the urgent need to develop a thorough understanding of the ecological dynamics of phytoplankton in drinking water reservoirs, as well as the synergistic impacts of eutrophication and climate change, to ensure the sustainability and safety of drinking water resources.

Phytoplankton, as the primary producers, rapidly and directly respond to changes in aquatic environments and are thus considered a vital biological indicator (Sun et al. 2023). It is essential to elucidate how abiotic environments influence the distribution patterns of phytoplankton, affected by continuing climate change and anthropogenic activities, for the preservation of drinking water sources in the reservoir. Neutral‐based and niche‐based theories constitute two important and complementary frameworks for understanding community assembly (Behrenfeld and Bisson 2024; Chen et al. 2019; Litchman et al. 2012). The neutral theory suggests that communities are shaped by random processes, including birth, death, immigration, and dispersal (Behrenfeld and Bisson 2024). In contrast, niche‐based theory asserts that deterministic abiotic elements, like temperature and nutrients, in conjunction with biotic factors (e.g., the grazing pressure of zooplankton), are fundamental determinants of phytoplankton community structure (Litchman et al. 2012). Their driving forces frequently vary by region (Jin et al. 2022). Environmental factors significantly influence the structure of phytoplankton communities in reservoirs, owing to their relatively restricted dispersion capability (Jin et al. 2022). The recent resurgence of trait‐based ecological methods has enhanced niche theory by endeavoring to define species' ecological niches according to their traits (Litchman et al. 2012). Numerous studies have evaluated phytoplankton realized niches, including the mean and breadth of niches, by employing generalized additive models (GAMs) and species distribution models (SDM) based on phytoplankton abundance data observed through traditional microscopy (Edwards et al. 2013; Hernández Fariñas et al. 2015; Irwin et al. 2012) or phytoplankton pigment data derived from high‐performance liquid chromatography (HPLC) combined with the CHEMTAX (HPLC‐CHEMTAX) approach (Xiao et al. 2019, 2018; Zhong et al. 2020). These studies have demonstrated that realized niches have yielded fresh insights into the impacts of environmental changes on the successions of phytoplankton communities. Furthermore, Wei et al. (2024) have shown that the realized niches of phytoplankton were critical to interpreting the dynamics of phytoplankton blooms and forecasting the responses of eutrophic lakes to the pressures of human activities and climate change. Nevertheless, the majority of research on phytoplankton community succession attributed to realized niches has predominantly focused on marine ecosystems (Irwin et al. 2012; Xiao et al. 2018; Zhong et al. 2020), while studies in freshwater environments remain insufficient (Wei et al. 2024).

There are several techniques for phytoplankton analysis, ranging from traditional microscopy to advanced methods such as HPLC coupled with CHEMTAX (HPLC‐CHEMTAX) for pigment‐based identification (Llorente et al. 2024; Yu et al. 2021), flow cytometry (Li et al. 2022), and high‐throughput sequencing (Tanttu et al. 2023). Each approach possesses distinct advantages and disadvantages. While the HPLC‐CHEMTAX method was unable to identify specific phytoplankton species, it effectively encompasses all phytoplankton size classes, which is often challenging to detect using microscopy or flow cytometry (Liu et al. 2019; Zhong et al. 2022a). Moreover, this approach is a chemical analysis technique that is more efficient, comparable, and repeatable than other methods. For instance, microscopic examinations depend on highly skilled experts in phytoplankton taxonomy (Yu et al. 2021), and high‐throughput sequencing often faces challenges such as limited quantitative accuracy, relatively high technical costs, and complex data analysis requirements. In contrast, the chemical analysis technique offers a more standardized and objective framework, providing consistent results across different studies and laboratories. This makes it extensively employed in marine research (Xiao et al. 2019, 2018; Zhong et al. 2022a), and several freshwater systems, such as lakes (Anil et al. 2015; Schlüter et al. 2016) and reservoirs (Yu et al. 2021).

Shanmei (SM) Reservoir as a drinking water source is the largest reservoir in Fujian Province, Southeast China. Increasing human activities such as agricultural fertilization, livestock farming, and sewage discharge have led to significant nitrogen pollution, resulting in a high nitrogen‐to‐phosphorus ratio (Liu et al. 2019). The SM Reservoir is currently classified as mesotrophic, but it shows signs of progressing toward eutrophication, posing a threat to water quality (Meng et al. 2022; Xu et al. 2024). However, there has been limited research on the phytoplankton community structure in the SM Reservoir, and their influencing factors remain unclear. In this study, we aimed to investigate the spatiotemporal variations in the phytoplankton community of the SM Reservoir and identify the underlying factors driving these changes. We hypothesize that the succession patterns and dynamics of phytoplankton communities in a drinking water reservoir are primarily explained by their realized niche characteristics in response to key environmental factors. This approach also seeks to explore how phytoplankton communities respond to the pressures of anthropogenic activities and global warming. Our findings emphasize the importance of realized niches in understanding phytoplankton dynamics, offering valuable insights for the scientific management and prevention of phytoplankton blooms in reservoirs.

## 2 Materials and Methods

### 2.1 Sampling Information

**FIGURE 1:**
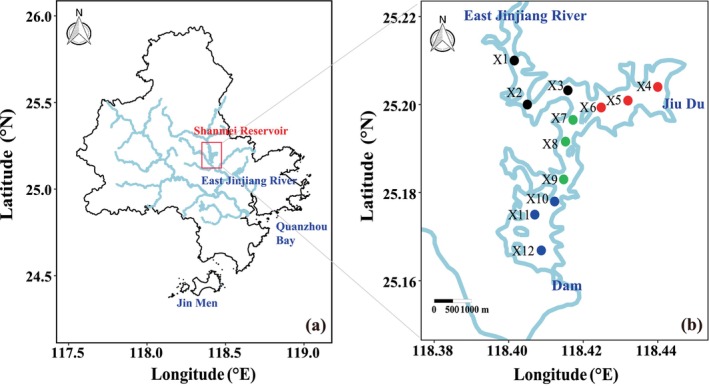
Location of Shanmei Reservoir (a) and the sampling stations in the Shanmei Reservoir (b). Black, red, green, and blue points correspond to the sampling stations located at the reservoir entrance, exit, center, and dam of SM Reservoir, respectively.

The study area is situated in the SM Reservoir, located along the Jinjiang River in Southeast China (Figure 1). The SM reservoir spans 26 km^2^, with a total storage capacity of 655 million m^3^ and a maximum depth of approximately 50 m (Figure 1). It serves multiple functions, including power generation, irrigation, and water supply (Liu et al. 2019), and provides drinking water for over 6 million residents in Quanzhou and Jinmen City. Therefore, investigating changes in the phytoplankton community structure of the reservoir is crucial for ensuring the safety of its water quality. Seven field observations were then carried out between 2022 to 2024, covering observations in August 2022, November 2022, February 2023, April 2023, August 2023, November 2023, and January 2024. We defined December, January, and February as winter; March through May as spring; June through August as summer; and September through November as autumn. Sampling was conducted at 12 stations: Stations X1, X2, and X3 were located at the reservoir entrance, Stations X4, X5, and X6 at the exit, Stations X7, X8, and X9 in the center, and Stations X10, X11, and X12 at the dam of SM Reservoir (Figure 1b). Surface water samples were taken from all sites, while vertical samples were collected from key monitoring Stations X2, X5, X9, and X12 to explore the vertical dynamics of nutrients and the phytoplankton community in the water column.

### 2.2 Physicochemical Variables

Water temperature, pH, dissolved oxygen (DO), and conductivity were measured on‐site using the HACH portable multi‐parameter water quality analyzer HQ440d. Nutrient and pigment samples were collected with a water sampler and kept in a portable freezer box for transport to the laboratory. Nutrient samples were filtered through Whatman GF/F membranes and stored at −20°C. Nitrate (NO_3_—N), nitrite (NO_2_—N), dissolved reactive phosphorus (DRP), and dissolved silicate (DSi) were analyzed using a QuAAtro nutrient flow analyzer, with detection limits of 0.006 μmol/L, 0.001 μmol/L, 0.019 μmol/L, and 0.03 μmol/L, respectively. NO_X_ was defined as the combined concentrations of NO_3_—N and NO_2_‐N.

### 2.3 Biological Analysis

200–600 mL water was filtered through 25‐mm GF/F filters (Whatman) for the extraction of phytoplankton pigments and thereafter stored at −80°C until analysis. Phytoplankton pigments were quantified via HPLC following extraction with N, N‐dimethylformamide and ammonium acetate (Zhong et al. 2020). The concentrations of pigments were quantified based on criteria produced by the Danish Hydraulic Institute (DHI) Water and Environment, Hørsholm, Denmark. Five phytoplankton groups—Bacillariophyta (Baci), Chlorophyta (Chlo), Cryptophyta (Cryp), Cyanophyta (Cyan), and Pyrrophyta (Pyrr) − were determined using CHEMTAX V1.95 based on ten diagnostic pigments: peridinin, fucoxanthin, violaxanthin, alloxanthin, lutein, neoxanthin, zeaxanthin, Chlorophyll b, diadinoxanthin, echinenone, and total Chlorophyll *a* (TChl *a*) (Anil et al. 2015; Schlüter et al. 2016). The initial input ratio matrix was modified according to the research conducted by Yu et al. (2021) and Schlüter et al. (2016), with both the initial and final matrices displayed in Table S1. To get accurate values of phytoplankton groups, we carried out 10 consecutive runs of CHEMTAX, using the output ratio matrix from each run as the input for the following run (Latasa 2007).

Phytoplankton samples were collected in accordance with the guidelines in the National Environmental Standards of the People's Republic of China, specifically the “Determination of Phytoplankton in Water‐Microscopic Counting Method using 0.1 mL Counting Chamber” (HJ 1216–2021). A 500 mL water sample was taken in the field and preserved with 1.5% Lugol iodine solution. In the laboratory, the samples were left to settle for at least 48 h, then concentrated to 30 mL for microscopic analyses (Federation and Association 2005). A 0.1 mL aliquot of the concentrated sample was placed into a 0.1 mL (20 × 20 mm) counting chamber and enumerated under an optical microscope (400 × magnification, Jiang'nan, China) in triplicate. Six phytoplankton groups−Pyrrophyta (Pyrr), Bacillariophyta (Baci), Cyanophyta (Cyan), Chlorophyta (Chlo), Euglenophyta (Eugl), and Cryptophyta (Cryp) − were identified, and their abundances (cells/L) were calculated using Equation (1) (Casamayor et al. 2000).

(1) $$N=\frac{A}{\mathrm{Ac}}\times \frac{V1}{V0}\times \frac{n}{V}\times 1000$$

In this equation, *N* represents the phytoplankton abundance in cells per liter of the water sample. A and Ac refer to the areas of the counting chamber (mm^2^) and the counting areas (mm^2^), respectively. V0 and V1 are the volumes of the original water sample (500 mL) and the concentrated sample (30 mL), respectively. V denotes the volume of the counting chamber (0.1 mL), and n is the number of phytoplankton counted within the defined counting area (cells).

The concentrations of phytoplankton groups calculated via CHEMTAX with both the concentrations of marker pigments and the abundances of phytoplankton groups determined through microscopic analysis were further compared in order to validate the pigment‐based phytoplankton compositions results (Figures S1, S2). There were strong, positive correlations between the concentrations of phytoplankton groups and their respective marker pigments (Figure S1), confirming that the marker pigments used were suitable in this study. Furthermore, a scatterplot comparing phytoplankton concentrations determined by the HPLC‐CHEMTAX method with abundances from microscopic analysis demonstrated a strong agreement between the two approaches (Figure S2). It further validated the efficacy of the pigment‐based chemotaxonomy method for determining phytoplankton communities.

### 2.4 Statistical Analyses

All statistical analyses were performed using R version 4.2.4 (Core 2014). The realized niches of five phytoplankton groups, including their mean realized niche (*μ*) and niche breadth (*σ*), were estimated using a combination of maximum entropy (MaxEnt) and generalized additive models (GAMs), following the method described by Zhong et al. (2020). Canonical correspondence analysis (CCA) and principal component analysis (PCA) were conducted using the “Vegan” package. CCA was employed to assess the effect of environmental factors on the succession of phytoplankton communities in the SM Reservoir, while PCA was employed to investigate the relationships between the five phytoplankton groups and their realized niches in response to environmental factors.

## 3 Results

### 3.1 Spatiotemporal Variations in Physicochemical Factors

In the surface water of the SM Reservoir, physicochemical variables exhibited significant seasonal variations (Table S1). Temperature ranged from 18.30°C to 32.80°C, with the highest values recorded in summer. Values of pH, DO, and conductivity ranged from 6.61 to 10.43, from 5.46 to 12.69 mg/L, and 109.10 to 172.40 μs/cm, respectively. The NO_X_ concentrations varied from 21.59 to 164.45 μmol/L, with NO_3_—N constituting the majority of NO_X_. Concentrations of DRP and DSi ranged from 0.01 to 0.23 μmol/L and from 32.68 to 247.60 μmol/L, respectively. Furthermore, the NO_X_/DRP molar ratios were relatively high (> 268), indicating that the surface water was phosphate‐deficient.

**FIGURE 2:**
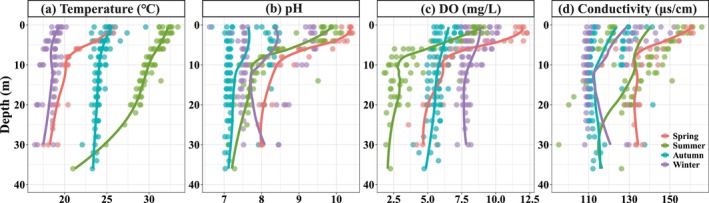
The vertical variations of temperature (a), pH (b), DO (c), and conductivity (d) in the water column in spring, summer, autumn, and winter. The colored lines are smoothed using the loess method based on the vertical data of Stations X2, X5, X9, and X12.

**FIGURE 3:**
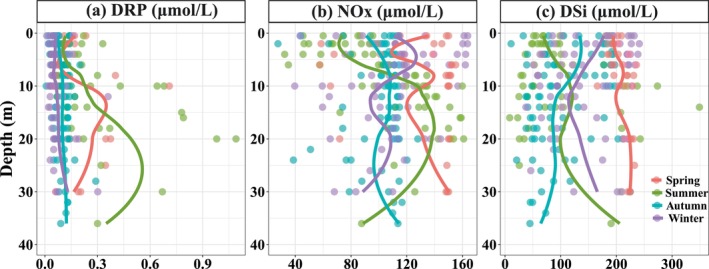
The vertical variations of DRP (a), NO_x_ (b), and DSi (c) in the water column in spring, summer, autumn, and winter. The colored lines are smoothed using the loess method based on the vertical data of Stations X2, X5, X9, and X12.

In the vertical water of the SM Reservoir, the distribution patterns of physicochemical parameters varied significantly (Figure 2). Temperature exhibited a clear seasonal pattern, with good mixing observed in autumn and winter. In contrast, pronounced thermoclines were present during the summer observations, and weak stratification was observed in spring. During periods of stratification, pH and DO values changed dramatically at depths of 5–10 m (Figure 2). For nutrient concentrations in the water column (Figure 3), NO_X_ concentrations exhibited obvious changes across water layers in different seasons. The vertical distribution patterns of DRP showed no obvious difference across layers in autumn and winter, while its concentrations initially increased with water depth and then decreased in spring and summer. The patterns of DSi showed significant differences between seasons, representing that the maximum occurred in spring, followed by winter and summer, and the minimum was found in autumn.

### 3.2 Dynamics of Phytoplankton Communities in the Drinking Reservoir

**FIGURE 4:**
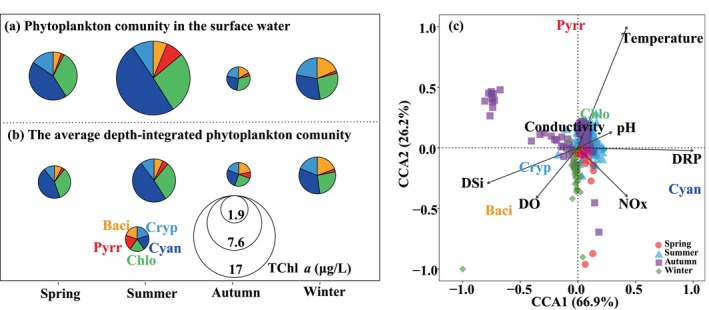
Variations of surface (a) and average depth‐integrated (b) phytoplankton compositions in the vertical water in spring, summer, autumn, and winter in the SM Reservoir; canonical correspondence analysis (CCA) of the relationship between the phytoplankton community and physicochemical factors (c).

Phytoplankton biomass and community composition showed significant variability (Figure 4 and Figure S3), with TChl *a* concentrations ranging from 252 to 24,008 ng/L throughout the water column of the SM reservoir. During spring, surface TChl *a* concentrations varied from 3120 to 14,354 ng/L, with Cyanophyta, Chlorophyta, and Cryptophyta accounting for 38.2%–50.5%, 31.5%–36.6%, and 7.6%–21.4% of TChl *a* concentrations, respectively. The average depth‐integrated phytoplankton biomass was 3304 ng/L, and Cyanophyta, Chlorophyta and Cryptophyta accounted for averaged 45%, 34%, and 11% of TChl *a* concentrations, respectively (Figure 4b). In the water column, Cryptophyta dropped with increasing water depth, but concentrations of Cyanophyta, Chlorophyta, and Bacillariophyta initially rose with water depth, subsequently declined, and then grew again at the bottom layer. Their maximums occurred at a depth of 5 m (Figure S3a). Cyanophyta consisted mostly of *Pseuanabaena* and *Microcystis*, while Chlorophyta were predominantly represented by *Chlamydomonas*, *Chlorella*, and *Scenedesmus*, respectively. Cryptophyta primarily consisted of *Cryptomonas*. During summer, the surface and average depth‐integrated TChl *a* concentrations varied from 4828 to 23,188 ng/L and 1716 to 11,145 ng/L, respectively. The predominant phytoplankton groups were Cyanophyta and Chlorophyta, which accounted for 33.4%–60.0% and 25.0%–38.0% of average depth‐integrated TChl *a* concentrations, respectively (Figure 4b). Phytoplankton concentrations generally decreased with increasing water depth (Figure S3b). Their predominant Cyanophyta species were *Pseuanabaena, Raphidiopsis*, and *Spirulina*. Chlorophyta were dominated by *Scenedesmus* and *Cosmarium*. During autumn, phytoplankton concentrations were comparatively low, with average depth‐integrated TChl *a* concentrations varying from 935 to 3069 ng/L (Figure 4b). Phytoplankton community was mainly composed of Chlorophyta, Cyanophyta, Bacillariophyta, and Cryptophyta, with Bacillariophyta and Cryptophyta reaching the highest proportions at 45.0% and 30.3%, respectively. The predominant Bacillariophyta species were *Melosira* and *Synedra*, whereas Cryptophyta were primarily represented by *Chroomonas acuta* and *Cryptomonas ovata* . Phytoplankton groups concentrations exhibited little fluctuations throughout the water column (Figure S3c). During winter, surface and average depth‐integrated TChl *a* concentrations varied from 1677 to 19,100 ng/L, and from 2368 to 5268 ng/L, respectively (Figure 4a). Bacillariophyta and Cryptophyta exhibited elevated proportions during winter (Figure 4b). Except for Pyrrophyta, other phytoplankton groups demonstrated comparable concentrations through the water column, with vertical patterns resembling those observed in spring (Figure S3d).

### 3.3 The Effect of Physicochemical Parameters on the Phytoplankton Communities

The relationship between physicochemical variables and phytoplankton compositions was performed by CCA (Figure 4c). Environmental parameters accounted for 17.6% of the variance in phytoplankton community of the SM reservoir. Temperature, conductivity, and pH exhibited significant positive correlations with Chlorophyta, indicating their links with its proliferation. Cryptophyta and Bacillariophyta had a strong correlation with DSi and DO, while showing a negative correlation with water temperature, DRP, and NOx. Cyanophyta, conversely, were associated with DRP and NOx.

**FIGURE 5:**
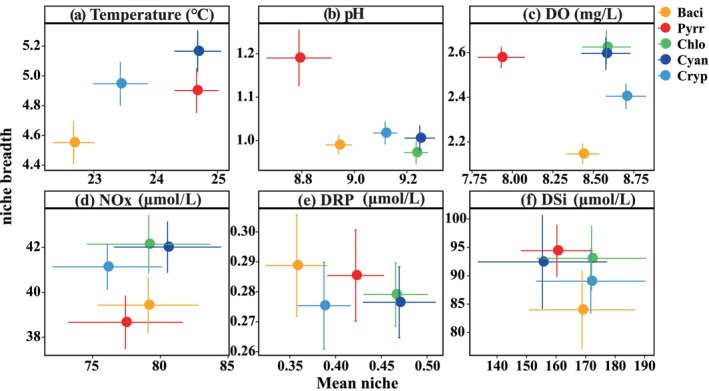
Mean and breadth of the realized niches for five phytoplankton groups in temperature (a), pH (b), DO (c), NOx (d), DRP (e), and DSi (f) in the SM reservoir. Error bars indicate the 95% confidence interval on each parameter from 100 bootstrapped resampling.

**FIGURE 6:**
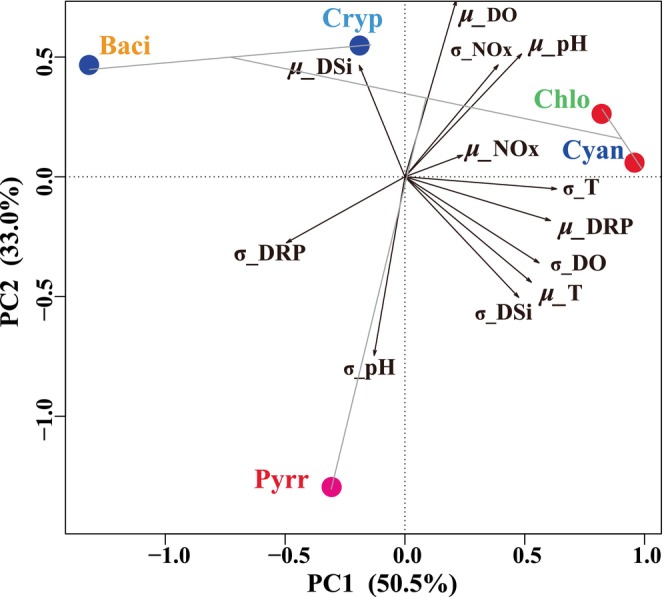
Principle component analysis for mean niches (μ) and niche breadth (σ) of phytoplankton in the SM reservoir. Cluster analysis was based on the Euclidean distances among the standardized values of the first two PCA components, and the number of clusters (the blue, red, and pink points represent three clusters) were determined by the scree plots.

For better understanding the effect of these environmental variables on the phytoplankton community, realized niches of phytoplankton, including the mean niche and niche breadths, were assessed (Figure 5). Cyanophyta and Chlorophyta demonstrated analogous realized niche patterns, both exhibiting high mean temperature niches, with values reaching 24.7°C. These groups also showed relatively high mean niches for pH, DO, NOx, and DRP, whereas Cyanophyta have a lower DSi niche in contrast to Chlorophyta. Pyrrophyta were clearly defined by a relatively high mean temperature niche, low mean pH and DO niches, and intermediate nutrient niches, differentiating them from other phytoplankton groups. Bacillariophyta and Cryptophyta exhibited relatively low mean temperature, intermediate pH, and high DO niches, alongside wide niche breadths for nutrients. PCA was performed to elucidate the distribution patterns of phytoplankton based on the realized niches for temperature, pH, DO, and nutrient concentrations (Figure 6). The first two principal components explained 83.5% of the variance in realized traits, categorizing phytoplankton into three groups. Pyrrophyta formed a distinct group associated with wide pH and DRP niches. Another group, comprising Chlorophyta and Cyanophyta, was mainly characterized by high mean temperature, pH, DO, NOx, and DRP niches. A third group, comprising Bacillariophyta and Cryptophyta, was more closely aligned with the mean DSi niche and distanced from the mean temperature and DRP niches.

## 4 Discussion

### 4.1 Realized Niches Explain the Dynamics of Phytoplankton Communities

Variations in phytoplankton were influenced by the environmental parameters including temperature, pH, DO, and nutrient concentrations, as demonstrated by the CCA analysis (Figure 4c). The recent application of realized niche theory to phytoplankton has enhanced mechanistic comprehension of phytoplankton community dynamics. Phytoplankton display biological variety, characterized by essential niche dimensions including environmental resources like temperature, DO, and nutrients, as well as pressures such as zooplankton grazing (Litchman et al. 2012). Measuring these traits is frequently arduous and laborious. To address this issue, Irwin et al. (2012) have proposed that phytoplankton niche traits be estimated from large long‐term field observation data in natural environments. However, most research has been predominantly focused on marine ecosystems (Irwin et al. 2012; Litchman et al. 2012; Xiao et al. 2019; Zhong et al. 2020), with comparatively fewer studies in freshwater ecosystems (Edwards et al. 2013; Wei et al. 2024). In our study, we assessed the niche traits of phytoplankton in the reservoir, including the mean niche and niche width, based on field observation using methods similar to those applied in the Taiwan Strait (Zhong et al. 2020). Our findings have indicated that realized niches contributed greatly to the understanding of phytoplankton dynamics in a drinking water reservoir.

The mean niche and niche breadth of phytoplankton indicate their adaptability to the environment. The niche breadth of phytoplankton correlates with their sensitivity to environmental variables; a broader niche enhances their flexibility, whereas a narrower niche suggests increased sensitivity to environmental changes. The response of Pyrrophyta to pH and DRP elucidated the ecological importance of niche breadth. Pyrrophyta have a wide niche breadth in both pH and DRP, indicating a significant tolerance to variations in these environmental factors. Previous studies have reported that Pyrrophyta can produce alkaline phosphatase to hydrolyze soluble organic phosphorus into inorganic forms (Zhong et al. 2022b), suggesting their reduced reliance on inorganic phosphorus. Chlorophyta and Cyanophyta exhibited niche overlaps, characterized by high mean temperature, pH, DO, and NOx niches, along with relatively wide niche breadths in these parameters. The high concentrations of Chlorophyta and Cyanophyta across all seasons (Figure 4) may be attributed to their broad niches in temperature and nutrients of NOx and DRP. This was further supported by observations in the Xipi Reservoir, where the abundance of Chlorophyta and Cyanophyta increased with rising temperature and pH (Zhong et al. 2022b). Chlorophyta thrive in hyper‐eutrophic environments linked to higher temperatures (Zhang et al. 2023b). Cyanophyta also flourish under relatively high temperature circumstances, frequently resulting in blooms during the summer. This phenomenon has been noted in other large lakes in China, including Taihu, Chaohu, and Dianchi, where Cyanophyta blooms are particularly prevalent during this season (Wang et al. 2023). Cyanophyta in the SM reservoir were primarily composed of *Pseudanabaena* and *Microcystis* (Figure 5). Several studies have shown that bloom‐forming cyanobacteria, such as *Microcystis*, attain peak growth rates at relatively high temperatures, frequently above 25°C (Huisman et al. 2018). *Pseudanabaena* in lakes and reservoirs often has higher densities throughout the summer and autumn seasons when water temperatures range from 20°C to 30°C, which is favorable for its growth (Huang et al. 2018). Cyanophyta flourish under eutrophic environments (Lu et al. 2023), particularly where nitrogen concentrations exceeded 1.5 mg/L (Wei et al. 2024), which is further verified by their relatively high mean NOx niche (Figures 5 and 6). Furthermore, most cyanobacteria prefer alkaline environments (Fang et al. 2018), with *Pseudanabaena* exhibiting optimum growth at a pH of around 11 (Gao et al. 2018), underscoring its adaptation to high pH niches.

Bacillariophyta and Cryptophyta have low‐temperature and high‐DSi niches (Figures 5 and 6), aligning with their relatively high abundance in the water column during winter (Figure 4b and Figure S3). Their prevalence under low‐temperature conditions has been documented in the Xipi reservoir (Zhong et al. 2022b). Many studies have shown that Bacillariophyta are predominantly found in environments characterized by low temperatures and high turbulence (Zhong et al. 2020, 2022b). Bacillariophyta utilize silicate for the synthesis of their siliceous shells and show positive relationships with DSi, with low DSi concentrations inhibiting their growth (Ha et al. 2022). Similarly, Cryptophyta thrive in tropical lakes with deep water mixing, low light conditions, and abundant nutrient availability (Tanttu et al. 2023). Their flagella and phycobiliproteins facilitate vertical migration in water and optimize light absorption (Qu et al. 2024; Tanttu et al. 2023). Cryptophyta populations are constrained by ammonium nitrogen, and their numbers commence to decline when ammonia nitrogen concentrations fall to 0.1 mg/L (Qu et al. 2024). Furthermore, Cryptophyta constitute a significant portion of bacterivores, serving an essential function in the transference of carbon from prokaryotes to higher trophic levels (Grujcic et al. 2018).

Under the multiple pressures of climate change and anthropogenic activities, the realized niches of phytoplankton are valuable for further exploring the succession of phytoplankton communities. Cryptophyta and Bacillariophyta, which are characterized by lower mean temperatures, showed their heightened vulnerability to global warming. Similar findings have been observed in ocean systems (Wang et al. 2024). Conversely, Chlorophyta and Cyanophyta would prosper in environments with rising water temperatures and increasing nutrient concentrations. This is particularly true for buoyant cyanobacteria such as *Microcystis*, *Anabaena*, and *Pseudanabaena*, which are well‐suited to the stratified water layers formed by higher water temperatures.

### 4.2 Management Implications

The physicochemical parameters in SM Reservoir exhibited significant seasonal variability. The pH values ranged from 6.61 to 10.43, with exceedances of standard limits predominantly occurring during spring and summer. This trend is likely driven by the substantial proliferation of phytoplankton during these seasons, which leads to considerable consumption of dissolved CO_2_, consequently elevating pH levels. Additionally, phosphorus concentrations remained relatively low; however, nitrogen concentrations were usually far above the eutrophication thresholds. This resulted in the high NO_X_/DRP molar ratios (> 268, Table S1), indicating a phosphate‐deficient condition in the reservoir. Phosphorus was a limiting factor for phytoplankton growth in SM Reservoir and, to some extent, influenced the proliferation of phytoplankton. This was supported by the findings of Xu et al. (2024), and further revealed that manganese inhibited the vertical transport of phosphorus, consequently reducing the potential for phytoplankton bloom.

Cyanophyta and Chlorophyta were the dominant phytoplankton groups in SM reservoir, especially during spring and summer, which is validated by microscopic analyses and HPLC‐CHEMTAX method (Figures S1 and S2). Cyanophyta consisted mainly of *Pseuanabaena*, *Raphidiopsis*, and *Microcystis*, and most of them could release odor‐causing compounds (Guo et al. 2024; Hooper et al. 2023). Numerous studies have shown that the growth and metabolism of filamentous cyanobacteria, such as *Pseudanabaena*, *Oscillatoria*, *Planktothrix*, and *Aphanizomenon*, can produce odor‐causing compounds, including 2‐methylisoborneol and geosmin (Guo et al. 2024; Wu et al. 2024). Among these, *Pseudanabaena* is the most prolific producer of such metabolites (Hooper et al. 2023). These compounds impart a distinct earthy/musty odor to drinking water sources, thereby compromising both the aquatic environment and the quality of water supply in reservoirs. This may explain the distinct earthy odor observed in fish from the reservoir during late spring and early summer. These findings further highlight the need to focus on the realized niche characteristics of *Pseudanabaena*, as well as the pathways and mechanisms by which it produces odor‐causing compounds.

Furthermore, through the analysis of niche characteristics, the mean niche and niche breadth of phytoplankton under various environmental parameters can be quantitatively evaluated, providing valuable insights for the prevention and control of phytoplankton blooms. For instance, Cyanophyta exhibit higher mean niches in NOx and DRP, with a mean temperature niche of 24.7°C. This implies that when the temperature approaches 24.7°C, the probability of Cyanophyta blooms increases significantly under sufficient nutrient conditions. While this study calculated the realized niches of broad categories of phytoplankton, future research focusing on the niches of specific bloom‐forming species, particularly *Pseudanabaena* as a key producer of odor‐causing compounds, across different environmental variables would be more beneficial for the prevention and control of phytoplankton blooms, as well as ensuring the sustainable supply of drinking water.

## 5 Conclusions

**FIGURE 7:**
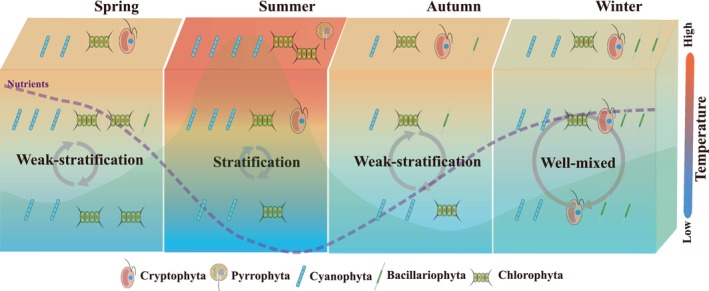
Conceptual structure of the distribution patterns of phytoplankton community in the SM reservoir. The background color represents the phytoplankton biomass.

This study demonstrated that the TChl *a* concentrations in the SM Reservoir ranged from 252 to 24,008 ng/L, peaking in summer, followed by spring and winter, with the lowest in autumn. Chlorophyta and Cyanophyta were the dominant phyla throughout the year, but the proportions of Cryptophyta and Bacillariophyta to TChl *a* concentrations were significantly higher in autumn and winter (Figure 7). The realized niches in water temperature, pH, DO, and nutrient concentrations contribute to explaining the distribution patterns of the phytoplankton community. Under the pressures of global changes and human activities, Chlorophyta and Cyanophyta are likely to thrive due to their realized niche characteristics, which include relatively high mean temperature and nutrient niches. In contrast, Cryptophyta and Bacillariophyta, which have lower mean temperature niches, would exhibit declining trends. Furthermore, incorporating the niche characteristics of specific phytoplankton species would further aid in the prevention and control of phytoplankton blooms in drinking reservoirs, ultimately improving the management and safety of the drinking water resources.

## Author Contributions

**Shuhua Wang:** funding acquisition (equal), investigation (equal), writing – review and editing (equal). **Xujun Liang:** data curation (equal), investigation (equal), writing – review and editing (equal). **Shanshan Zhang:** data curation (equal), funding acquisition (equal), investigation (equal), writing – review and editing (equal). **Mingjiang Cai:** investigation (equal), methodology (equal). **Zhangxian Xie:** investigation (equal). **Lizhen Lin:** methodology (equal). **Zhenguo Chen:** writing – review and editing (equal). **Yiyong Rao:** funding acquisition (equal), investigation (equal). **Yanping Zhong:** conceptualization (equal), funding acquisition (equal), investigation (equal), methodology (equal), project administration (equal), supervision (equal), validation (equal), visualization (equal), writing – original draft (equal), writing – review and editing (equal).

## Conflicts of Interest

The authors declare no conflicts of interest.

## Supporting information


Data S1.

## Data Availability

Data will be made available on https://osf.io/m9t8z/.

## References

[ece371180-bib-0001] Anil, A. C. , M. Tamm , R. Freiberg , I. Tõnno , P. Nõges , and T. Nõges . 2015. “Pigment‐Based Chemotaxonomy—A Quick Alternative to Determine Algal Assemblages in Large Shallow Eutrophic Lake?” PLoS One 10, no. 3: e0122526. 25803038 10.1371/journal.pone.0122526 PMC4372291

[ece371180-bib-0002] Behrenfeld, M. J. , and K. M. Bisson . 2024. “Neutral Theory and Plankton Biodiversity.” Annual Review of Marine Science 16: 283–305. 10.1146/annurev-marine-112122-105229 37368954

[ece371180-bib-0003] Cai, G. , Y. Ge , Z. Dong , et al. 2024. “Temporal Shifts in the Phytoplankton Network in a Large Eutrophic Shallow Freshwater Lake Subjected to Major Environmental Changes due to Human Interventions.” Water Research 261: 122054. 38986279 10.1016/j.watres.2024.122054

[ece371180-bib-0004] Casamayor, E. O. , H. Schafer , L. Baneras , C. Pedros‐Alio , and G. Muyzer . 2000. “Identification of and Spatio‐Temporal Differences Between Microbial Assemblages From Two Neighboring Sulfurous Lakes: Comparison by Microscopy and Denaturing Gradient Gel Electrophoresis.” Applied and Environmental Microbiology 66, no. 2: 499–508. 10653710 10.1128/aem.66.2.499-508.2000 PMC91855

[ece371180-bib-0005] Chen, W. , K. Ren , A. Isabwe , H. Chen , M. Liu , and J. Yang . 2019. “Stochastic Processes Shape Microeukaryotic Community Assembly in a Subtropical River Across Wet and Dry Seasons.” Microbiome 7, no. 1: 1–16. 31640783 10.1186/s40168-019-0749-8 PMC6806580

[ece371180-bib-0006] Core, T. R. 2014. A Language and Environment for Statistical Computing. R foundation for statistical computing.

[ece371180-bib-0007] Edwards, K. F. , E. Litchman , and C. A. Klausmeier . 2013. “Functional Traits Explain Phytoplankton Responses to Environmental Gradients Across Lakes of the United States.” Ecology 94, no. 7: 1626–1635. 23951722 10.1890/12-1459.1

[ece371180-bib-0008] Fang, F. , Y. Gao , L. Gan , X. He , and L. Yang . 2018. “Effects of Different Initial pH and Irradiance Levels on Cyanobacterial Colonies From Lake Taihu, China.” Journal of Applied Phycology 30, no. 3: 1777–1793.

[ece371180-bib-0009] Federation, W.E., and Association, A.P.H . 2005. Standard Methods for the Examination of Water and Wastewater. American Public Health Association (APHA).

[ece371180-bib-0010] Gao, J. , J. Zhu , M. Wang , and W. Dong . 2018. “Dominance and Growth Factors of *Pseudanabaena Sp*. in Drinking Water Source Reservoirs, Southern China.” Sustainability 10, no. 11: 3936. 10.3390/su10113936.

[ece371180-bib-0011] Grujcic, V. , J. K. Nuy , M. M. Salcher , et al. 2018. “Cryptophyta as Major Bacterivores in Freshwater Summer Plankton.” ISME Journal 12, no. 7: 1668–1681. 29463895 10.1038/s41396-018-0057-5 PMC6018765

[ece371180-bib-0012] Guo, H. , R. Li , S. Xue , et al. 2024. “Considerable Declines in Odor in a Drinking Water Reservoir: Variations of Odorous Community, Precursor Enzymes Abundance, Distribution, and Environmental Dominant Factors.” Water Research 268: 122767. 39541849 10.1016/j.watres.2024.122767

[ece371180-bib-0013] Ha, X. , Y. Gao , J. Jia , et al. 2022. “The Role of Phytoplankton Communities on Coupled Carbon‐Silicon Cycling in a Large Floodplain Lake System.” Ecohydrology & Hydrobiology 22, no. 3: 408–419.

[ece371180-bib-0014] Hernández Fariñas, T. , C. Bacher , D. Soudant , C. Belin , and L. Barillé . 2015. “Assessing Phytoplankton Realized Niches Using a French National Phytoplankton Monitoring Network.” Estuarine, Coastal and Shelf Science 159: 15–27.

[ece371180-bib-0015] Hooper, A. S. , P. Kille , S. E. Watson , S. R. Christofides , and R. G. Perkins . 2023. “The Importance of Nutrient Ratios in Determining Elevations in Geosmin Synthase (geoA) and 2‐MIB Cyclase (Mic) Resulting in Taste and Odour Events.” Water Research 232: 119693. 36764104 10.1016/j.watres.2023.119693

[ece371180-bib-0016] Huang, X. , Z. Huang , X. P. Chen , et al. 2018. “The Predominant Phytoplankton of Pseudoanabaena Holding Specific Biosynthesis Gene‐Derived Occurrence of 2‐MIB in a Drinking Water Reservoir.” Environmental Science and Pollution Research International 25, no. 19: 19134–19142. 29725924 10.1007/s11356-018-2086-z

[ece371180-bib-0017] Huisman, J. , G. A. Codd , H. W. Paerl , B. W. Ibelings , J. M. H. Verspagen , and P. M. Visser . 2018. “Cyanobacterial Blooms.” Nature Reviews. Microbiology 16, no. 8: 471–483. 29946124 10.1038/s41579-018-0040-1

[ece371180-bib-0018] Irwin, A. J. , A. M. Nelles , and Z. V. Finkel . 2012. “Phytoplankton Niches Estimated From Field Data.” Limnology and Oceanography 57, no. 3: 787–797.

[ece371180-bib-0019] Jin, L. , H. Chen , Y. Xue , J. Soininen , and J. Yang . 2022. “The Scale‐Dependence of Spatial Distribution of Reservoir Plankton Communities in Subtropical and Tropical China.” Science of the Total Environment 845: 157179. 35809738 10.1016/j.scitotenv.2022.157179

[ece371180-bib-0020] Latasa, M. 2007. “Improving Estimations of Phytoplankton Class Abundances Using CHEMTAX.” Marine Ecology Progress Series 329: 13–21.

[ece371180-bib-0021] Li, C. , K. P. Chiang , E. A. Laws , et al. 2022. “Quasi‐Antiphase Diel Patterns of Abundance and Cell Size/Biomass of Picophytoplankton in the Oligotrophic Ocean.” Geophysical Research Letters 49, no. 5: e2022GL097753.

[ece371180-bib-0022] Litchman, E. , K. F. Edwards , C. A. Klausmeier , and M. K. Thomas . 2012. “Phytoplankton Niches, Traits and Eco‐Evolutionary Responses to Global Environmental Change.” Marine Ecology Progress Series 470: 235–248.

[ece371180-bib-0023] Liu, M. , X. Chen , Y. Chen , L. Gao , and H. Deng . 2019. “Nitrogen Retention Effects Under Reservoir Regulation at Multiple Time Scales in a Subtropical River Basin.” Waternb 11, no. 8: 1685.

[ece371180-bib-0024] Llorente, A. , H. Fraile , B. Gartzia de Bikuña , and S. Seoane . 2024. “HPLC Validation as a Management Tool in Artificial Water Storage Ponds.” Limnologica 105: 126160.

[ece371180-bib-0025] Lu, Y. , Y. Tuo , L. Zhang , et al. 2023. “Vertical Distribution Rules and Factors Influencing Phytoplankton in Front of a Drinking Water Reservoir Outlet.” Science of the Total Environment 902: 166512. 37619726 10.1016/j.scitotenv.2023.166512

[ece371180-bib-0026] Meng, H. , J. Zhang , and Z. Zheng . 2022. “Retrieving Inland Reservoir Water Quality Parameters Using Landsat 8‐9 OLI and Sentinel‐2 MSI Sensors With Empirical Multivariate Regression.” International Journal of Environmental Research and Public Health 19, no. 13: 7725. 35805386 10.3390/ijerph19137725 PMC9265597

[ece371180-bib-0027] Qu, F. , Y. Wang , D. Yu , and N. Chen . 2024. “High‐Frequency Monitoring Reveals Phytoplankton Succession Patterns and the Role of Cryptophyte in a Subtropical River Reservoir.” Algal Research 82: 103680. 10.1016/j.algal.2024.103680.

[ece371180-bib-0028] Ruan, Q. , H. Liu , Z. Dai , F. Wang , and W. Cao . 2024. “Damming Exacerbates the Discontinuities of Phytoplankton in a Subtropical River in China.” Journal of Environmental Management 351: 119832. 38128215 10.1016/j.jenvman.2023.119832

[ece371180-bib-0029] Schlüter, L. , S. Behl , M. Striebel , and H. Stibor . 2016. “Comparing Microscopic Counts and Pigment Analyses in 46 Phytoplankton Communities From Lakes of Different Trophic State.” Freshwater Biology 61, no. 10: 1627–1639.

[ece371180-bib-0030] Song, C. , C. Fan , J. Zhu , et al. 2022. “A Comprehensive Geospatial Database of Nearly 100 000 Reservoirs in China.” Earth System Science Data 14, no. 9: 4017–4034.

[ece371180-bib-0031] Sun, K. , W. Deng , J. Jia , and Y. Gao . 2023. “Spatiotemporal Patterns and Drivers of Phytoplankton Primary Productivity in China's Lakes and Reservoirs at a National Scale.” Global and Planetary Change 228: 104215.

[ece371180-bib-0032] Tanttu, H. , D. Verschuren , W. De Crop , et al. 2023. “High‐Throughput Sequencing and Marker Pigment Analysis of Freshwater Phytoplankton: A Direct Comparison With Microscopic Count Data in the Tropical Crater Lakes of Western Uganda.” Limnologica 99: 126052.

[ece371180-bib-0033] Wang, C. , S. Cai , Z. Tong , et al. 2024. “Unveiling Differential Thermal Sensitivities in Marine Phytoplankton Within the China Seas.” Limnology and Oceanography Letters 9, no. 5: 583–592. 10.1002/lol2.10411.

[ece371180-bib-0034] Wang, S. , X. Zhang , C. Wang , and N. Chen . 2023. “Multivariable Integrated Risk Assessment for Cyanobacterial Blooms in Eutrophic Lakes and Its Spatiotemporal Characteristics.” Water Research 228: 119367. 36417795 10.1016/j.watres.2022.119367

[ece371180-bib-0035] Wei, Q. , Y. Xu , and A. Ruan . 2024. “Spatial and Temporal Patterns of Phytoplankton Community Succession and Characteristics of Realized Niches in Lake Taihu, China.” Environmental Research 243: 117896. 38081348 10.1016/j.envres.2023.117896

[ece371180-bib-0036] Wu, D. , M. Chen , A. Shen , and Y. Shi . 2024. “Spatiotemporal Dynamics of 2‐Methylisoborneol Produced by Filamentous Cyanobacteria and Associated Driving Factors in Lake Taihu, China.” Harmful Algae 138: 102703. 39244238 10.1016/j.hal.2024.102703

[ece371180-bib-0037] Xiao, W. , E. A. Laws , Y. Xie , et al. 2019. “Responses of Marine Phytoplankton Communities to Environmental Changes: New Insights From a Niche Classification Scheme.” Water Research 166: 115070. 31525510 10.1016/j.watres.2019.115070

[ece371180-bib-0038] Xiao, W. , L. Wang , E. Laws , et al. 2018. “Realized Niches Explain Spatial Gradients in Seasonal Abundance of Phytoplankton Groups in the South China Sea.” Progress in Oceanography 162: 223–239.

[ece371180-bib-0039] Xu, Z. , L. Ge , W. Zou , et al. 2024. “The Underestimated Role of Manganese in Modulating the Nutrient Structure in a Eutrophic Seasonally‐Stratified Reservoir.” Water Research 260: 121940. 38885556 10.1016/j.watres.2024.121940

[ece371180-bib-0040] Yu, X. , J. R. Yang , J. Chen , A. Isabwe , and J. Yang . 2021. “On the Use of Chemotaxonomy, a Phytoplankton Identification and Quantification Method Based on Pigment for Quick Surveys of Subtropical Reservoirs.” Environmental Science and Pollution Research International 28, no. 3: 3544–3555. 32920686 10.1007/s11356-020-10704-4

[ece371180-bib-0042] Zhang, H. , Y. Yang , X. Liu , et al. 2023b. “Novel Insights in Seasonal Dynamics and Co‐Existence Patterns of Phytoplankton and Micro‐Eukaryotes in Drinking Water Reservoir, Northwest China: DNA Data and Ecological Model.” Science of the Total Environment 857, no. 1: 159160. 36195142 10.1016/j.scitotenv.2022.159160

[ece371180-bib-0043] Zhang, Y. L. , J. M. Deng , Y. Q. Zhou , et al. 2024. “Drinking Water Safety Improvement and Future Challenge of Lakes and Eservoirs.” Science Bulletin 69, no. 22: 3558–3570. 38955563 10.1016/j.scib.2024.06.018

[ece371180-bib-0044] Zhong, Y. , E. A. Laws , J. Zhuang , et al. 2022a. “Responses of Phytoplankton Communities Driven by Differences of Source Water Intrusions in the El Niño and La Niña Events in the Taiwan Strait During the Early Spring.” Frontiers in Marine Science 9: 997591. 10.3389/fmars.2022.997591.

[ece371180-bib-0045] Zhong, Y. , X. Liu , W. Xiao , et al. 2020. “Phytoplankton Community Patterns in the Taiwan Strait Match the Characteristics of Their Realized Niches.” Progress in Oceanography 186: 102366.

[ece371180-bib-0046] Zhong, Y. , Y. Su , D. Zhang , et al. 2022b. “The Spatiotemporal Variations in Microalgae Communities in Vertical Waters of a Subtropical Reservoir.” Journal of Environmental Management 317: 115379. 35751236 10.1016/j.jenvman.2022.115379

