# Supplementary material for: Dynamics of Phytoplankton Communities and Their Characteristics of Realized Niches in a Drinking Reservoir
Source: Ecol Evol. 2025 Apr 11;15(4):e71180. doi: 10.1002/ece3.71180 (PMC11991924; doi:10.1002/ece3.71180)
Supplement: Supplementary file 1 — Data S1. [file ECE3-15-e71180-s001.docx]

**Supplementary**

**Table S1 Initial (a) and final (b) ratio pigment: chlorophyll *a* ratio matrices ^a^**

| **Class** | **perid** | **fucox** | **dindino** | **neox** | **violax** | **allox** | **lutein** | **zeax** | **echi** | **chl_b** | **TChl*a*** |
| --- | --- | --- | --- | --- | --- | --- | --- | --- | --- | --- | --- |
| 1. Initial ratios | | | | | | | | | | | |
| Baci | 0 | 0.585 | 0.093 | 0 | 0 | 0 | 0 | 0.001 | 0 | 0 | 1 |
| Chlo | 0 | 0 | 0 | 0.138 | 0.134 | 0 | 0.154 | 0.001 | 0 | 0.369 | 1 |
| Cyan | 0 | 0 | 0 | 0 | 0 | 0 | 0 | 0.112 | 0.038 | 0 | 1 |
| Pyrr | 0.33 | 0 | 0.019 | 0 | 0 | 0 | 0 | 0 | 0 | 0 | 1 |
| Cryp | 0 | 0 | 0 | 0 | 0 | 0.582 | 0 | 0 | 0 | 0 | 1 |
| 1. Final ratios | | | | | | | | | | | |
| Baci | 0 | 0.452 | 0.110 | 0 | 0 | 0 | 0 | 0.001 | 0 | 0 | 0 |
| Chlo | 0 | 0 | 0 | 0.015 | 0.023 | 0 | 0.095 | 0.001 | 0 | 0.266 | 0 |
| Cyan | 0 | 0 | 0 | 0 | 0 | 0 | 0 | 0.053 | 0.057 | 0 | 0 |
| Pyrr | 0.245 | 0 | 0.014 | 0 | 0 | 0 | 0 | 0 | 0 | 0 | 0.245 |
| Cryp | 0 | 0 | 0 | 0 | 0 | 0.368 | 0 | 0 | 0 | 0 | 0 |

^a^ Baci, Chlo, Cyan, Pyrr, Cryp, perid, fucox, dindino, neox, violax, allox, lutein, zeax, echi, chl_b, and TChl*a* were Bacillariophyta, Chlorophyta, Cryptophyta, Cyanophyta, Pyrrophyta, peridinin, fucoxanthin, diadinoxanthin, neoxanthin, violaxanthin, alloxanthin, lutein, zeaxanthin, echinenone, chlorophyll b, and total Chlorophyll *a*, respectively.

**Table S2 Summary of physicochemical variables in the Shanmei Reservoir ^a^**

| **Sampling**  **time** | **Temperature**  **(**°C**)** | **pH** | **DO**  **(mg/L)** | **Conductivity**  **(****μs/cm)** | **NO_2_-N**  **(μmol/L)** | **NO_3_-N**  **(μmol/L)** | **NO_X_**  **(μmol/L)** | **DRP**  **(μmol/L)** | **DSi**  **(μmol/L)** |
| --- | --- | --- | --- | --- | --- | --- | --- | --- | --- |
| 3/8/2022 | 32.04±0.57 | 9.65±0.06 | 8.48±0.28 | 121.83±1.12 | 1.64±0.62 | 58.39±23.60 | 60.03±24.22 | 0.07±0.02 | 75.27±17.76 |
| 10/11/2022 | 25.98±1.24 | 8.07±0.44 | 7.04±0.23 | 132.73±7.03 | 0.10±0.04 | 95.67±15.71 | 95.76±15.72 | 0.05±0.03 | 183.46±7.01 |
| 18/2/2023 | 19.03±0.37 | 9.24±0.48 | 10.01±0.48 | 143.48±11.45 | 2.68±1.37 | 134.15±34.86 | 136.84±36.09 | 0.06±0.02 | 223.04±18.66 |
| 18/4/2023 | 25.15±0.54 | 10.33±0.09 | 12.09±0.38 | 159.00±3.33 | 5.93±1.56 | 118.02±31.91 | 123.94±33.41 | 0.17±0.04 | 196.64±12.56 |
| 17/8/2023 | 32.35±0.90 | 9.89±0.06 | 8.86±0.20 | 159.38±5.23 | 1.10±0.03 | 89.92±0.42 | 91.02±6.45 | 0.18±0.06 | 77.09±36.34 |
| 21/11/2023 | 24.80±0.88 | 7.05±0.26 | 5.79±0.27 | 113.18±0.92 | 0.21±0.12 | 94.98±34.22 | 95.19±34.28 | 0.10±0.04 | 87.20±42.55 |
| 15/1/2024 | 19.73±0.37 | 7.31±0.47 | 7.77±0.23 | 117.78±12.43 | 0.41±0.19 | 89.62±33.30 | 90.03±33.39 | 0.07±0.07 | 124.44±43.50 |

^a^ values are mean ± standard division. DO, NO_2_-N, NO_3_-N, NO_X_, DRP, DSi are dissolved oxygen, nitrite, nitrate, the sum of nitrate and nitrite, dissolved reactive phosphorus, and dissolved silicate, respectively.


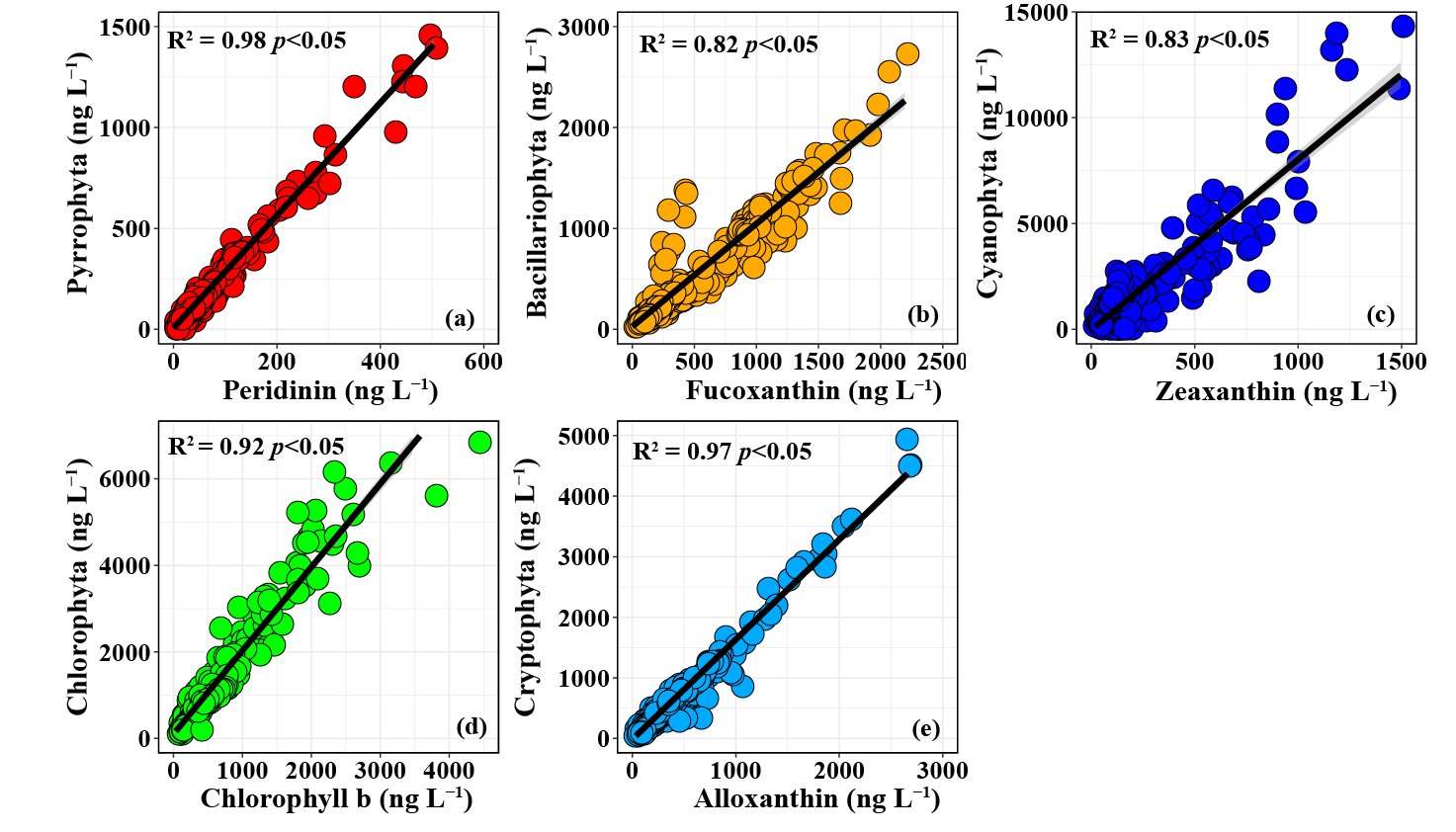


**FIGURE S1 Correlations between concentrations of marker pigments and concentrations of phytoplankton groups estimated from CHEMTAX. (a) peridinin vs Pyrrophyta; (b) Fucoxanthin vs Bacillariophyta; (c) Zeaxanthin vs Cyanophyta; (d)Chlorophyll b vs Chlorophyta and (e)Alloxanthin vs Cryptophyta.**

**
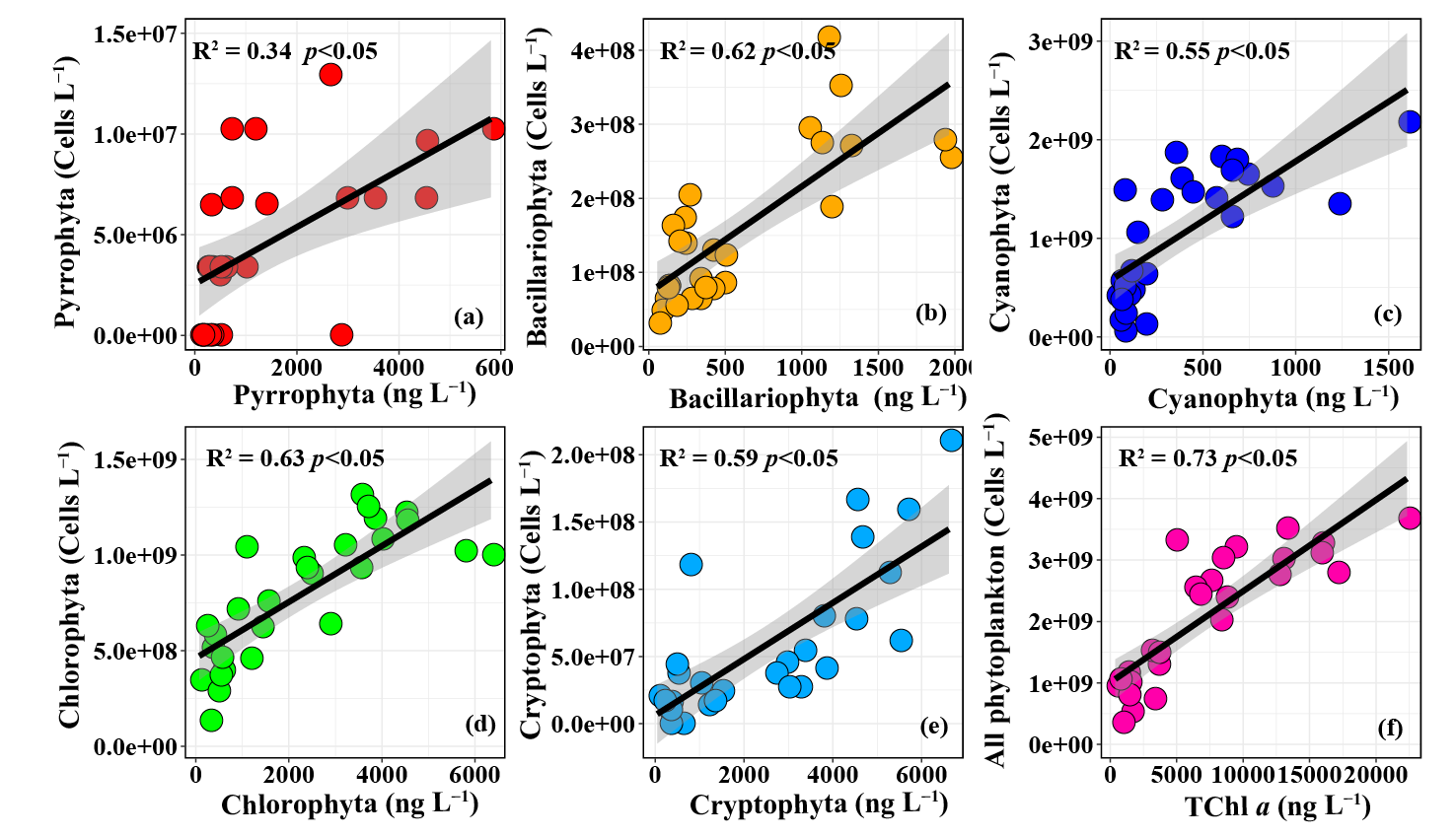
**

**FIGURE S2 Comparisons between phytoplankton groups determined via HPLC-CHEMTAX and microscopic analyses. (a) Pyrrophyta; (b) Bacillariophyta; (c) Cyanophyta; (d) Chlorophyta; (e) Cryptophyta and (f) total chlorophyll *a* vs all phytoplankton abundance.**


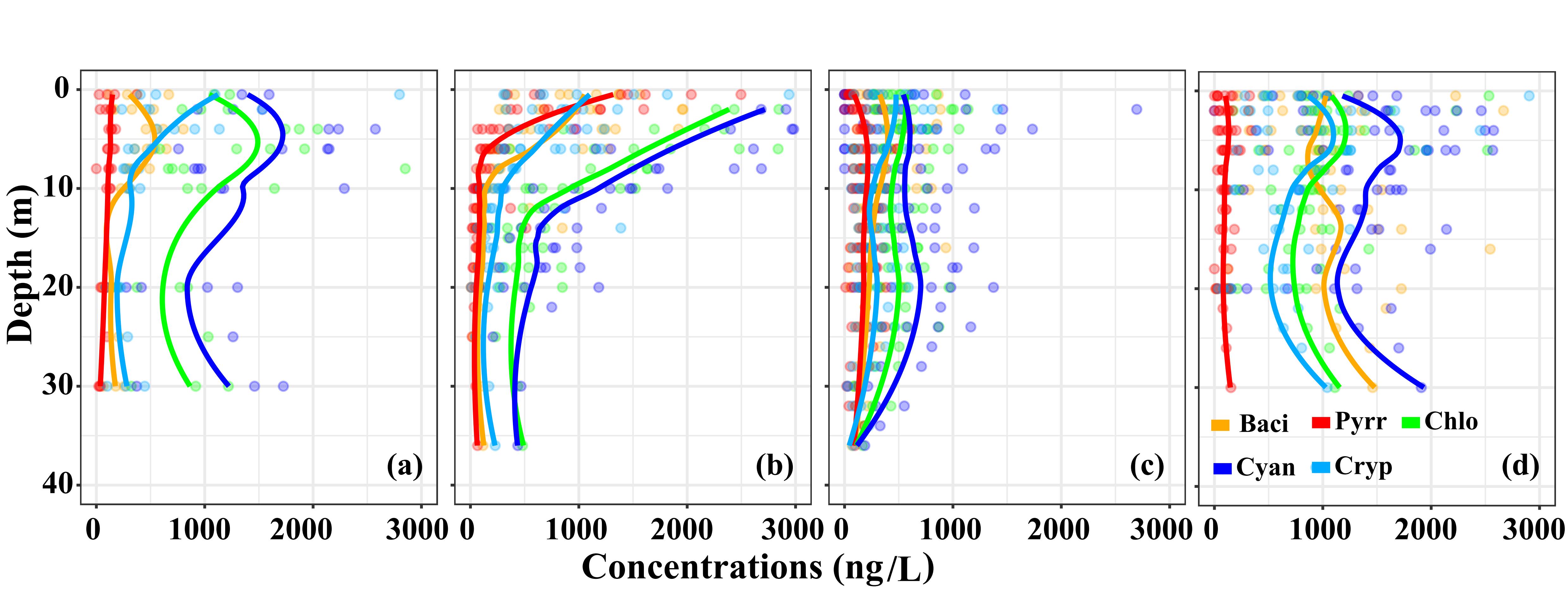


**FIGURE S3 The vertical variations of phytoplankton community in the water column in spring (a), summer (b), autumn (c), and winter (d). The colored lines are smoothed using the loess method based on the vertical data of Stations X2, X5, X9, and X12**
